# Tomato Plant-Associated Xanthoderma: Case Report and Review of Exogenous Causes of Yellow Skin

**DOI:** 10.7759/cureus.47218

**Published:** 2023-10-17

**Authors:** Philip R Cohen

**Affiliations:** 1 Dermatology, University of California Davis School of Medicine, Sacramento, USA; 2 Dermatology, Touro College of Osteopathic Medicine, Vallejo, USA

**Keywords:** yellow, xanthotricosis, xanthoderma, trichomes, tomato, skin, plant, nails, hair, exogenous

## Abstract

The skin, hair, and nails can all present with yellow discoloration secondary to exogenous etiologies. Xanthoderma, yellow discoloration of the skin, can occur not only from exogenous sources secondary to topical contact with various substances but also from endogenous causes such as diseases from the liver and kidney, or oral medications. A 64-year-old man developed asymptomatic, yellow staining of his distal left forearm, hand, and fingertips. He was not receiving antimalarials, did not have hepatic or renal dysfunction, and had not applied any sunless tanning solutions to his skin. Prior to the appearance of his xanthoderma, he had been tending to a tomato plant in his yard; the yellow staining appeared on the areas of his left upper extremity that had contacted the stems and leaves of the tomato plant. Within two days, the yellow skin discoloration resolved spontaneously after several washings of the affected areas with soap and water. Tomato plants have trichomes that appear as hair-like structures on the stems and produce an oily substance; the trichomes not only produce the scent of the plant, but also provide protection from cold, drought, disease, and pests. Initially, when the oily substance contacts the skin, the skin appears yellow; subsequently, the skin may become black. The skin that has been stained by a tomato plant is referred to as "tomato skin" (TOMASK). In addition to reviewing the etiology of exogenous xanthoderma, this paper also summarizes the causes of exogenous yellow hair and yellow nails. Exogenous yellowing of the skin can result from various topical causes. Common topical etiologies of xanthoderma include not only contact with tomato plants, but also sunless tanning solutions (that contain dihydroxyacetone) and tobacco (that not only causes yellow staining of the white hair on men’s upper lip referred to as "smoker’s mustache", but also yellow staining of the nail plate and fingertips used to hold the cigarette or cigar). In summary, tomato plant-associated xanthoderma is a benign exogenous etiology of yellow staining of the skin which eventually resolves after several washings of the affected sites with soap and water.

## Introduction

Xanthoderma refers to yellow discoloration of the skin. Cutaneous yellow discoloration has also been described as carotenoderma (caused by eating an excessive amount of carrots or foods that are high in carotene content), jaundice (caused by liver disease-associated hyperbilirubinemia), and uremic pallor (caused by kidney disease). Systemic conditions and oral antimalarial medications such as quinacrine and mepacrine can cause yellow discoloration of the skin. In addition, people can develop yellow skin lesions such as sebaceous adnexal tumors and hyperlipidemia-associated xanthomas [1-3].

The surface of some plants contains trichomes; these are appendages that protect the plant from cold, drought, disease, and pests. Tomato plants have trichomes that appear as tiny hair-like structures and very small vesicles on the stem. "Tomato skin" may occur when the essential oils from the trichomes contact the skin, initially presenting as yellow discoloration that masks the normal skin color [4-6].

Tomato skin is an example of xanthoderma resulting from an exogenous etiology. In this case report, a man with yellow discoloration of his left wrist, hands, and fingertips after contact with the stem and leaves of a tomato plant will be described. In addition to tomato plant-associated yellow skin discoloration, other etiologies of exogenous xanthoderma are reviewed.

## Case presentation

**Figure 1:**
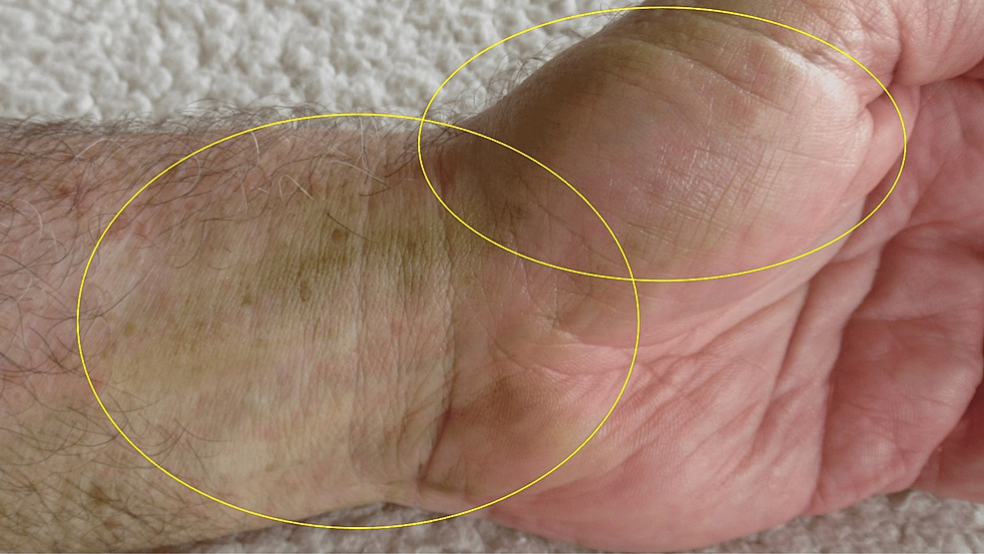
Tomato plant-associated xanthoderma on distal left forearm and left hand There is yellow discoloration (within yellow ovals) of the flexor wrist and the hypothenar eminence of the left upper extremity of a 64-year-old Caucasian man.

A 64-year-old Caucasian man, without any liver or kidney disease, presented with asymptomatic yellow discoloration of his left wrist, hand, and fingertips (Figure 1). No new medications had been initiated and he was not taking antimalarials. He had not applied any sunless tanning solutions to his skin.

**Figure 2:**
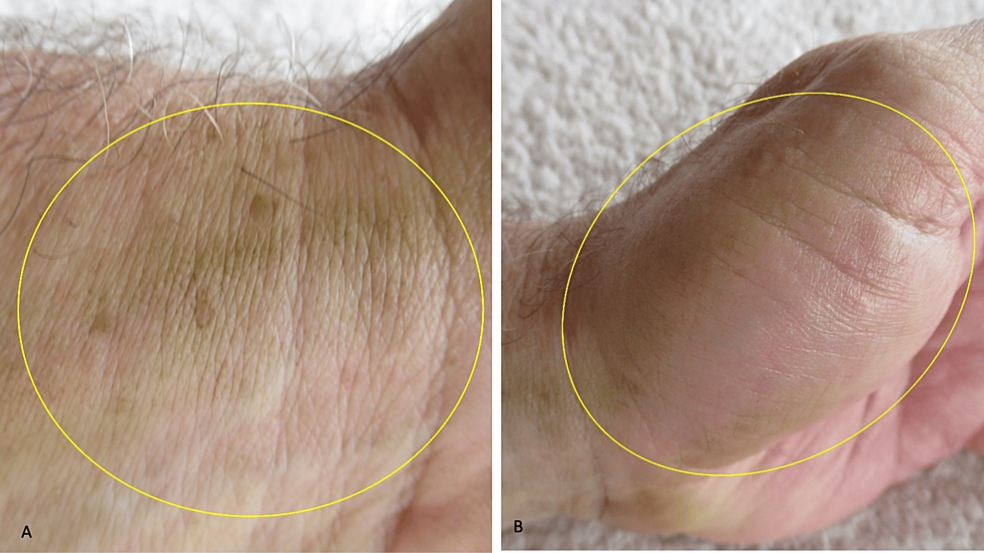
Yellow discoloration caused by contact with a tomato plant Closer images of the left flexor wrist (A) and the left hypothenar eminence (B) show xanthoderma (within the yellow ovals).

**Figure 3:**
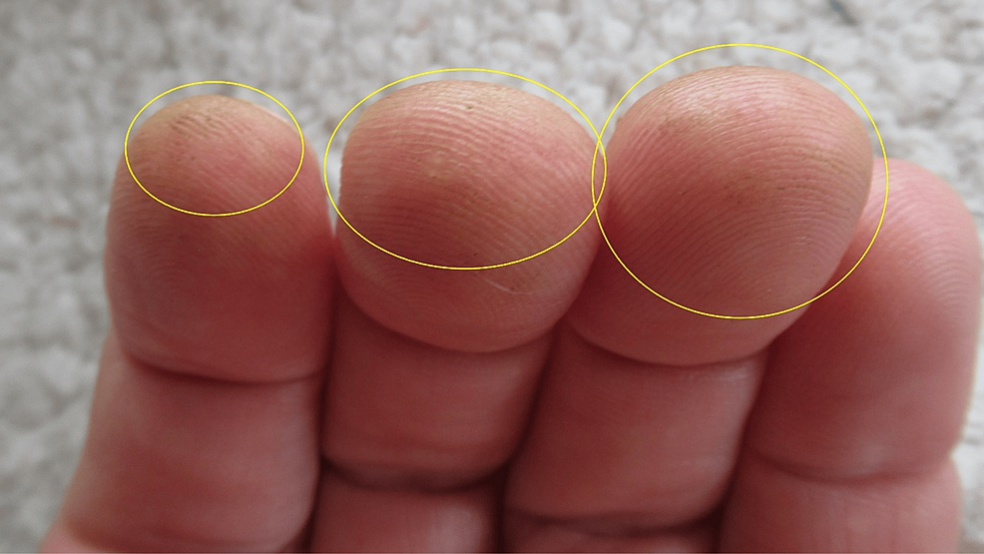
Tomato plant-related xanthoderma on the fingertips of the left hand The fingertips of the second, third, and fourth fingers on the left hand show yellow staining (within the yellow ovals) after having contacted the stem and leaves of a tomato plant.

**Figure 4:**
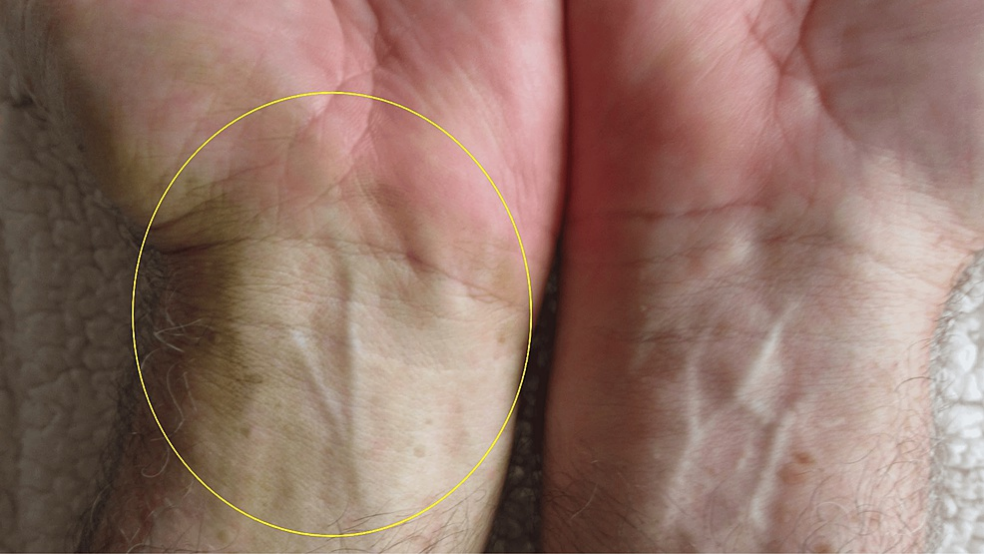
Comparison of tomato plant-associated yellow-stained skin and normal-appearing skin Xanthoderma is present on the left wrist appearing as yellow-stained skin (surrounded by a yellow oval); the man’s right wrist is unstained.

**Figure 5:**
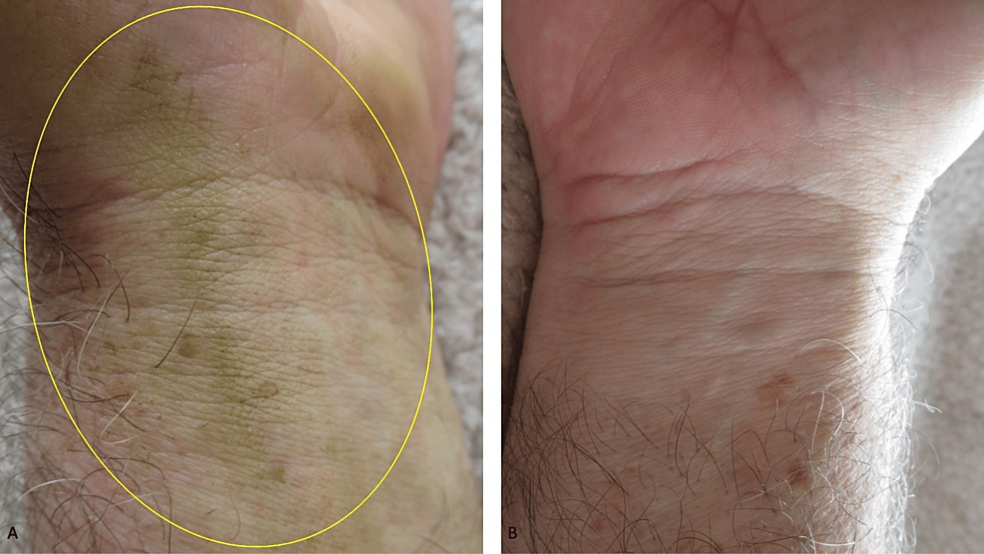
Closer image comparing the stained left wrist and the unstained right wrist and hand The xanthoderma appears yellow on the skin affected by the tomato plant-related xanthoderma on the left wrist (surrounded by a yellow oval) (A); there is no staining on the right wrist (B).

A cutaneous examination of his distal upper extremities was performed. Yellow discoloration was noted on his flexor distal left forearm predominantly involving his wrist and his left hand primarily affecting the hypothenar eminence (Figure 2). The tips of his second, third, and fourth fingers on the left hand were also stained yellow (Figure 3). In comparison, his distal right forearm and wrist were normal in appearance (Figures 4-5).

**Figure 6:**
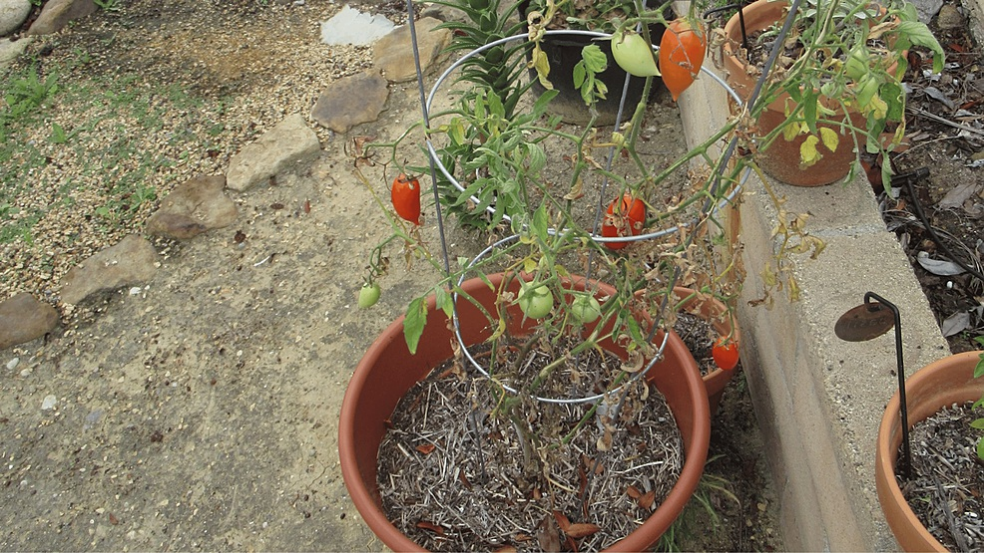
Tomato plant A tomato plant can cause exogenous xanthoderma after an individual contacts the trichomes that are located on the stems and leaves.

**Figure 7:**
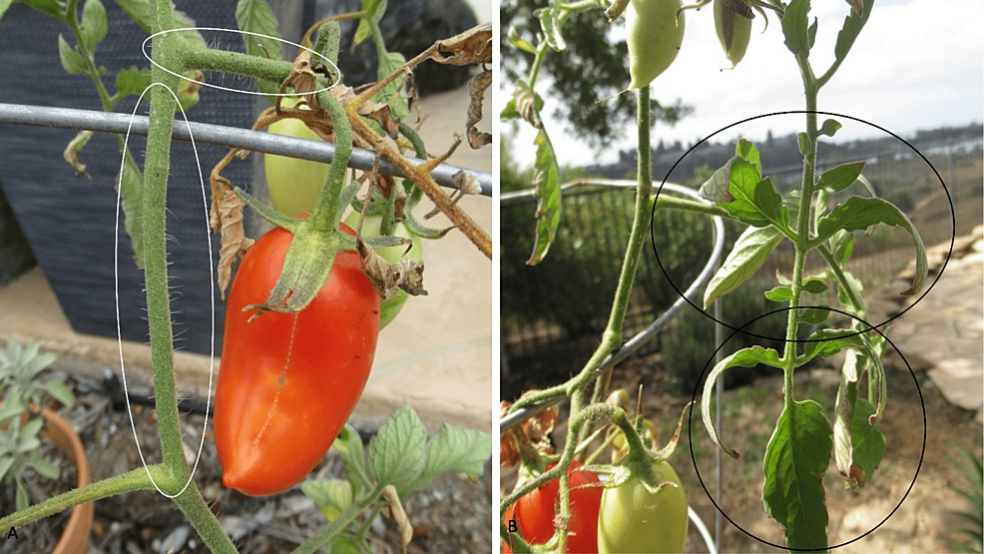
The stems and leaves of the tomato plant The hair-like structures on the stems (within the white ovals) of the tomato plant are trichomes (A). The trichomes also reduce the loss of plant heat and protect the plant tissues from ultraviolet light; in addition, the trichomes protect the plant from cold, drought, disease, and pests. The skin can also be stained yellow after contacting the leaves (within the black ovals) (B).

Additional history revealed that earlier that day - prior to the appearance of the yellow staining of his skin - he had been tending to a tomato plant in his yard (Figure 6). After the affected areas had contacted the stems and leaves of the tomato plant (Figure 7), he noticed the yellow staining of his skin. The yellow skin discoloration resolved spontaneously within the following two days after several washings of the affected areas with soap and water.

## Discussion

The skin developed yellow discoloration in the man described whose hands and fingers contacted the tomato plant. In addition, yellow discoloration can affect the cutaneous appendages such as the hair and nails. The etiology of these changes can be secondary not only to endogenous causes such as systemic conditions or medications but also to exogenous sources [1-18]. In addition to reviewing the etiology of exogenous xanthoderma, this paper also summarizes the causes of exogenous yellow hair and yellow nails.

Xanthotrichosis, or yellow hair, can result from yellow bacterial concretions most commonly on the axillary hair shaft and designated as trichobacteriosis, which was previously referred to as trichomycosis; it is caused by *Corynebacterium* species - such as *Corynebacterium tenuis* and *Corynebacterium flavenscens - *which are gram-positive diptheroids that colonize the affected hair [7]. Topical contact with numerous exogenous agents can result in yellow hair (Table 1) [2,3,8-13]. "Smoker’s mustache" refers to tobacco-associated yellow discoloration of upper lip hair in men who smoke cigarettes and cigars; it is more readily observed in men with light-colored hair or age-associated white hair [12].

**Table 1:** Exogenous causes of yellow hair Abbreviations:  CR, current report; MDA, 4,4’-methylenedianiline; MPD, 4,4’-metaphenylenediamine; Refs, references; w/v, weight in volume ^a^Yellow hair was observed when this was applied under acidic conditions. ^b^The curing agent Z consists of 32% w/v MDA and 44% w/v MPD. The curing agent Z, MDA, and MDP were used in the production of the fantip seal which is a molded epoxy-plastic structure that is utilized in the production of jet engines for use in civilian and military aircraft. ^c^A pesticide. ^d^This is a component of products used for sunless tanning. ^e^Yellowing of the hair occurred after exposure to selenium sulfide, with or without the application of bacitracin. ^f^Aromatic nitro compounds are yellow and used as dyes.

Etiology	Refs
Anthralin^a^	[8]
Curing agent Z^b^	[9,10]
Copper	[8]
Dinobuton^c^	[11]
Dihydroxyacetone^d^	[8]
Hydrogen peroxide	[8]
Hypochlorous acid	[8]
4,4’-Methylenedianiline (MDA)^b^	[9,10]
4,4’-Metaphenylenediamine (MPD)^b^	[9,10]
Minoxidil (1.5%)	[8]
1-naphthol derivatives	[8]
Picric acid	[8]
Resorcinol	[8]
Selenium sulfide^e^	[8]
Tar shampoo	[8]
Tobacco	[2,3,12]
Trinitrotoluene^f^	[11,13]

Yellow nails can be a component of yellow nail syndrome along with primary lymphedema and pulmonary disease which can include pleural effusion, recurrent pneumonia, or bronchiectasis [2,11]. Dermatophyte fungal infection of the nails can commonly present with yellow discoloration of the nail plate [2,11,14]. Oral tetracycline can result in yellow nail changes localized to the lunula [3].

Exogenous causes of yellow chromonychia are summarized in Table 2 [2,9-13,15-17]. Topical exposure to several pesticides has been associated with yellow discoloration of the nails; these include dinitro-ortho-cresol, dinobuton, diquat, and paraquat [11,13]. Tobacco can result in distal yellow staining of the smoker’s fingernails referred to as the "nicotine sign". "Harlequin nail" presents in prior smokers as the abrupt onset of normal proximal nail plate color with distal yellowing of the nail plate; when the length of the normal-appearing proximal nail is measured to the proximal nail fold, the clinician can estimate the duration of time - since fingernails grow approximately three millimeters each month - that the individual abruptly ceased smoking [3,12,15].

**Table 2:** Exogenous causes of yellow chromonychia Abbreviations: CR, current report; MDA, 4,4’-methylenedianiline; MPD, 4,4’-metaphenylenediamine; Refs, references; w/v, weight in volume ^a^The curing agent Z consists of 32% w/v MDA and 44% w/v MPD. The curing agent Z, MDA, and MDP were used in the production of the fantip seal which is a molded epoxy-plastic structure that is utilized in the production of jet engines for use in civilian and military aircraft. ^b^This is regarding industrial exposure to workers who handle the chromium salts. For example, calcium chromate is an odorless, yellow, crystalline (sand-like) powder used to inhibit corrosion and depolarize batteries and as a pigment. ^c^Aromatic nitro compounds are yellow and used as dyes. ^d^A pesticide. ^e^This was used in several steps during the process of amylase determination. ^f^This occurs most commonly with deep red nail polishes that contain D&C Reds No. 6, 7, 34, or 5 Lake; the staining of the nail occurs when the polish is dissolved instead of suspended. After one week of continuous wear, the nail plate will be stained yellow. Once the enamel has been removed, the stain will fade spontaneously in about two weeks. Only the nail surface has been stained, which can be confirmed by scraping the nail plate with a scalpel blade. ^g^Onycholysis and yellowish discoloration of the nail plate can be observed in flower handlers who develop rhus dermatitis from either poison ivy, poison oak, or poison sumac.

Etiology	Refs
Ascorbic acid preparation	[2,15]
Curing agent Z^a^	[9,10]
Chromium salts^b^	[11]
Dinitrobenzene^c^	[11,16]
Dinitrotoluene^c^	[11,16]
Dinitro-ortho-cresol (5%)^d^	[11]
Dinitro-salicylic acid^c,e^	[11,13]
Dinobuton^d^	[11]
Diquat^d^	[11]
4,4’-Methylenedianiline (MDA)^a^	[9,10]
4,4’-Metaphenylenediamine (MPD)^a^	[9,10]
Paraquat^d^	[11]
Red nail polish^f^	[2,17]
Rhus dermatitis^g^	[11]
Tobacco	[12,15]
Trinitrotoluene^c^	[11,13]

Yellow staining of the nails would not intuitively be considered a complication following the application of red nail polish; this has been observed when both the color is deep red (such as D&C Reds number 6, 7, and 34 or 5 Lake) and the polish pigment has been dissolved instead of suspended. Yellow staining of the nail plate will occur after seven days of continuously wearing the nail polish. The staining only affects the surface of the nail plate; it can be scraped off the nail with a scalpel blade. Once the enamel of the red polish has been removed, the yellow staining of the affected nail plate will spontaneously resolve in about 14 days [2,17].

In addition to antimalarials, endogenous causes of yellow skin may be observed in patients receiving other oral agents such as beta-carotene or antineoplastic drugs; the latter include sorafenib or sunitinib, whose ingestion results in thickened yellow hyperkeratosis of the palms and soles [2]. Over-ingestion of dipyridamole, a yellow compound, also resulted in xanthoderma [3]. Benzodiazepam-associated yellow staining of the cheeks and the soles was noticed two months after a 28-year-old woman began taking nitrazepam [13].

Exogenous causes of xanthoderma may result from topical contact with various agents (Table 3) [2-6,9,10,12,13,15,16,18]. Ink and henna tattoos can both result in yellow skin. The inoculation of cadmium pigment into the dermis creates permanent yellow tattoos and the application of yellow-stained henna results in temporary yellow staining of the skin which eventually spontaneously fades [2].

**Table 3:** Exogenous causes of yellow skin Abbreviations: CR, current report; DBNP, 2,6,-di-tert-butyl-4-nitrophenol; DBP, 2,6,-di-tert-butylphenol; MDA, 4,4’-methylenedianiline; MPD, 4,4’-metaphenylenediamine; Refs, references; w/v, weight in volume ^a^Curing agent Z consists of 32% w/v MDA and 44% w/v MPD. The curing agent Z, MDA, and MDP were used in the production of the fantip seal which is a molded epoxy-plastic structure that is utilized in the production of jet engines for use in civilian and military aircraft. ^b^This is a component of products used for sunless tanning. ^c^This was used in several steps during the process of amylase determination. ^d^DBP is an antioxidant additive in many synthetic lubricating oils and hydraulic fluids. An intensely yellow crystalline material, DBNP, is formed when the lubricating oil mist containing DBP passes through an electrostatic precipitator and is nitrated. Yellowing of the skin was noted by submarine personnel who came in contact with DBNP on their bedding, bulkheads and other surfaces of the submarine. ^e^Phenol is also known as carbolic acid; it can also be used as a disinfectant, chemical intermediate and for nail matrix ablation. Picric acid is a derivative of phenol. Both cause yellow staining on exposed skin and methemoglobinemia-associated blue discoloration of the lips and fingernails. ^f^Tomato plant associated-yellow xanthoderma is referred to as "tomato skin" (TOMASK); the acronym is derived from the first four letters of the word "tomato" and the first two letters of the word "skin".

Etiology	Refs
Ascorbic acid preparation	[2,15]
Cadmium tattoo	[2]
Curing agent Z^a^	[9,10]
Dihydroxyacetone^b^	[2,18]
Dinitro-salicylic acid^c^	[13]
2,6,-Di-tert-butyl-4-Nitrophenol (DBNP)^d^	[16]
Henna tattoo	[2]
4,4’-Methylenedianiline (MDA)^a^	[9,10]
4,4’-Metaphenylenediamine (MPD)^a^	[9,10]
Nitric acid (dilute)	[10]
Phenol^e^	[3]
Picric acid^e^	[3]
Tobacco	[3,12]
Tomato plants^f^	[4-6], CR
Trinitrophenylmethylnitramine (tetryl)	[10]

Topical ascorbic acid under occlusion has resulted in yellow discoloration of the hands and fingernails. As a treatment for hand eczema, a 56-year-old man immersed his hands for an hour twice daily in a homemade solution of analytical grade ascorbic acid (5-10%) with 5% glycerol weight in weight; thereafter, he would wrap the hands in a plastic film for 30 minutes. The following day, there would be yellow staining of not only the skin on his hands but also his fingernails; after he stopped using the preparation the yellow discoloration would spontaneously fade. The yellow discoloration of skin and nails recurred when he subsequently initiated the protocol again [15].

A study found that at least 54 workers in a factory dedicated to making molded plastics developed yellow staining of their skin, including their forearms and palms, their fingernails, and their hair. Molders accounted for 25 of the individuals; all the affected persons were involved in the production of the fantip seal which is a molded epoxy-plastic structure, measuring ten by ten centimeters, that is utilized in the production of jet engines in civilian and military aircraft. During the production of the molded plastic seal, all the affected workers were exposed to curing agent Z which consists of 32% weight in volume 4,4’-methylenedianiline (MDA) and 44% weight in volume 4,4’-methylenedianiline (MPD) [10,15]. 

Dihydroxyacetone causes a yellowing of the skin. Many sunless tanning preparations incorporate the three-carbon sugar moiety dihydroxyacetone as the active ingredient. Keratinizing stratum corneum contains an abundance of basic amino acids that dihydroxyacetone binds to form melanoidins; these are chromophores. Topically applied dihydroxyacetone only affects the stratum corneum; therefore, it does not have any ability to induce tanning in mucous membranes that lack a stratum corneum. Body sites characterized by thicker stratum corneum, such as the palms and soles acquire deeper tanning [18].

The degree of skin yellowing in individuals who apply sunless tanning preparations is influenced by the underlying skin tone. In individuals with lighter skin tones, Fitzpatrick type I (people who always burn and never tan) and Fitzpatrick type II (people who usually burn and minimally tan) the sunless-tanned skin treated with topical dihydroxyacetone will appear more yellow than in individuals with higher Fitzpatrick skin types. In addition, people with a ruddy complexion will appear more yellow after topical application of dihydroxyacetone [18].

Another investigation discovered that submarine crew members presented with yellow skin at the sites of contact with 2,6,-di-tert-butyl-4-nitrophenol (DBNP) on their bedding, bulkheads, and other surfaces of the submarine. DBNP is an intensely yellow crystalline material that is formed when the lubricating mist of 2,6,-di-tert-butylphenol (DBP) passes through an electrostatic precipitator and is nitrated. DBP is an antioxidant additive that was used in the synthetic lubricating oils and hydraulic fluids on the submarine [16].

Yellowing has also been observed at sites of direct contact with dilute nitric acid in the work environment. Dilute nitric acid is a colorless and transparent liquid that is used to make explosives, fertilizers, and dye. It changes color to yellow following decomposition which can occur not only at room temperature but also after exposure to direct sunlight and heat [5].

Trinitrophenylmethylnitramine (tetryl) can cause yellow stains on exposed skin in the work environment. It is an odorless, synthetic, yellow crystal-like powder that was used in the production of explosives mostly during World War I and World War II. In addition, it has been associated with causing liver cirrhosis [5].

Yellowing of the skin has occurred after exposure to either phenol or picric acid; however, since both compounds can cause methemoglobinemia, the patient’s lips and fingernails may have a blue discoloration. Phenol, also known as carbolic acid, could be a chemical intermediate, a disinfectant, or a reagent used to permanently ablate the matrix of nails. Picric acid is a derivative of phenol [3].

In several case reports, tobacco and tobacco byproducts resulted in yellow discoloration of the skin. Tobacco-associated skin discoloration is commonly an accompanying clinical feature in individuals with smoker’s mustache. In addition to xanthotrichia of the mustache hair, not only their fingernails but also the distal tips of their fingers may be stained yellow [12].

Cutaneous dyschromia may result from contacting the stem or leaves or both of tomato plants. Tomato plant-associated skin discoloration has been referred to as "tomato fingers" and "tomato hands". A more appropriate name is "tomato skin" (TOMASK); the acronym is derived from the first four letters of the word "tomato" and the first two letters of the word "skin" [5,6].

Trichomes are hair-like structures located on the stems of tomato plants; some appear as little bubbles covering the stem. They are also located on the leaves. Tomato plants have eight different trichomes: glandular trichomes (types I, IV, VI, and VII) and nonglandular trichomes (types II, III, V, and VIII) [4,6].

Trichomes originate from the outer epidermal cell tissue of the plant. Acyl flavonoids, terpenoids, and sugars are compounds present in the glands of type I, IV, and VI trichomes. They have a viscous oily consistency, provide the scent associated with the tomato plant, and can be clear or appear as a yellow secretion [4-6].

Trichomes have many essential roles for the tomato plant. They provide protection for the plant from cold, drought, disease, and pests; in addition, they reduce the loss of plant heat and protect the plant tissues from ultraviolet light. Their composition is influenced by the stresses encountered by the plant [4-6].

When the oily substance initially accumulates on the fingers and hands, it appears yellow. Subsequently, it may become black. After the color has changed, it has been referred to as tomato tar [5,6].

In this case report, the man developed yellow discoloration of his wrist, hypothenar eminence, and fingertips. The dyschromia was noticed immediately after these sites had come in contact with the stem and leaves of the tomato plant. The man’s yellow xanthoderma resolved after several washings of the sites with soap and water during the subsequent two days.

## Conclusions

Xanthoderma results not only from endogenous causes such as diseases from the liver and kidney, or oral medications, but also from topical contact with various substances. Common etiologies of exogenous xanthoderma include the application of dihydroxyacetone-containing sunless tanning solutions and tobacco-related yellow staining on the fingertips used to hold the cigarette or cigar. Yellow staining of the distal left forearm, hand, and fingertips of a 64-year-old man who neither smoked nor used sunless tanning solutions appeared on the areas of his left upper extremity that had contacted the stems and leaves of a tomato plant. Xanthochromia of tomato plant-stained skin is referred to as TOMASK. The yellow staining results from contacting the aromatic oily substance from trichomes. Trichomes are the hair-like structures on tomato plant stems that protect the plant from cold, drought, disease, and pests. The substance from the trichome initially appears yellow and may subsequently become black after contacting the skin. After several washings of the affected areas with soap and water the yellow skin discoloration of the man resolved spontaneously within two days. In summary, tomato plant-associated xanthoderma is a benign exogenous etiology of yellow staining of the skin which eventually resolves after repeated washings of the affected sites with soap and water.
